# The effectiveness of Tai Chi for patients with mild cognitive impairment: a systematic review and meta−analysis

**DOI:** 10.3389/fnins.2024.1467595

**Published:** 2024-11-25

**Authors:** Xinxin Shao, Yawei Xi, Lijie Pan, Xinru Li, Qianxin Lin, Keming Tian, Rui Wang, Yutong Gao, Hainan Gao, Zili Tan, Xiangyu Zhu

**Affiliations:** ^1^School of Acupuncture−Moxibustion and Tuina, Beijing University of Chinese Medicine, Beijing, China; ^2^Acupuncture and Rehabilitation Department, Liangxiang Hospital of Beijing Fangshan District, Beijing, China

**Keywords:** Tai Chi, mild cognitive impairment, cognitive function, memory, neuroplastic changes

## Abstract

**Objective:**

To explore the effectiveness of Tai Chi on cognitive function in patients with mild cognitive impairment (MCI).

**Methods:**

According to the PRISMA guidelines, randomized controlled trial (RCT) literature on the efficacy of Tai Chi on MCI patients was searched in China National Knowledge Network (CNKI), China Biomedical Literature Database (CBM), Wanfang Data, China Scientific Journal Database (VIP), PubMed, Embase, Duxiu Database, Web of Science and Cochrane Library from their inception to April 2024. The risk of bias in each study was appraised using the Cochrane risk−of−bias tool using Revman 5.4. Random effect model or fixed effect model was used to compare the effects of Tai Chi and control conditions on baseline and post−intervention assessment of cognitive function. Meta−analysis was performed using Stata15.0 software.

**Results:**

Nine studies fulfilled the inclusion criteria. Tai Chi significantly improved Montreal Cognitive Assessment (MoCA, SMD, 1.43, *p* < 0.00001), Delayed Recall Test (DRT, SMD, 0.90, *p* < 0.00001), verbal fluency test (VFT, SMD, 0.40, *p* < 0.00001), and Trail Making Test (TMT, SDM, −0.69, *p* < 0.00001) in MCI patients. Subgroup analyses showed that 24-forms Tai Chi was more effective than 8-forms Tai Chi in improving MoCA (SMD, 1.89, *p* < 0.00001) and 10-forms Tai Chi was more effective than 24-forms Tai Chi in improving DRT (SMD, 1.53, *p* < 0.00001).

**Conclusion:**

Tai Chi improved cognitive function in MCI patients, and Tai Chi types might be the influence factor on Tai Chi improving the global cognitive function and memory function in MCI patients.

**Systematic review registration:**

https://www.crd.york.ac.uk/prospero/.

## 1 Introduction

MCI has become a major public health issue worldwide (Chen et al., 2023). MCI refers to a neurodegenerative imbalance of brain functional networks that becomes more severe with age (Weijun, n.d.), which does not meet the diagnostic criteria for dementia. The clinical symptoms of MCI involve functional impairment in one or more cognitive domains, including attention, executive function, language, memory and learning, and visual/spatial function. MCI can affect individuals’ activities of daily living and social participation. Previous studies have confirmed that the incidence of dementia is higher among patients with MCI compared with the general population, and that MCI is a risk factor for the occurrence of dementia (Vos et al., 2015; Shen and Zhengping, 2024). The prevalence of MCI is more than 15% in the global population, with 10–20% for those aged ≥65 years (Bai et al., 2022), and the risk increases with age. Patients with MCI are at high risk for dementia, with about 5–17% of MCI patients diagnosed with clinical dementia each year. The global prevalence of dementia is expected to double every 20 years, from 46.8 million in 2015 to 131.5 million in 2050 (Owens et al., 2024). MCI and dementia are considered to constitute sequential phases of progression in the same disease spectrum. Early interventions in patients with MCI have been reported to delay the onset of dementia by an average of 5 years, reduce the number of dementia patients by 57%, and reduce annual medical insurance premiums by half (Wang et al., 2023; Li R. et al., 2022).

Various drug treatments for alleviating the symptoms of MCI are currently available, including ergot alkaloids, calcium antagonists (Petersen et al., 2018), ginkgo biloba extract (Kandiah et al., 2019), cholinesterase inhibitors (Reuben et al., 2024), and ion glutamate receptor antagonists. However, long-term use of these medications can lead to increased psychological and financial burdens on patients, as well as a higher risk of adverse reactions (Karssemeijer et al., 2017). Although acupuncture has been found to have clinical efficacy in treating MCI, some patients experience fear or fainting during acupuncture treatment, and compliance is often poor over time. Therefore, a growing number of studies have examined the potential beneficial effects of physical exercise in MCI (Biazus-Sehn et al., 2020). However, MCI occurs more frequently in older people than younger individuals, and the intensity of some types of physical exercise may be unsuitable for the older population. Additionally, the accessibility of some types of physical exercise is limited by the availability of suitable venues and equipment. Tai Chi could provide a low-intensity and accessible form of exercise that avoids these problems.

As a traditional Chinese rehabilitation exercise, Tai Chi involves the combination of exercise with breathing and mindfulness. Tai Chi emphasizes the harmonious balance of “body, breath and mind” (Rongman and Changxi, 2018), constituting a Chinese traditional mind–body exercise that is considered to be effective for recuperation of the “mind.” Previous research reported that Tai Chi was beneficial for the establishment of a network of connections among neurons to delay the degenerative decline of cranial nerves (Yaqiang and Feng, 2023). Evidence suggested that Tai Chi can improve cognitive functions and alleviate the accompanying symptoms of MCI in the elderly potentially by activating the expression of signals in different brain regions, altering their connectivity, increasing the brain volume, and modulating brain−derived neurotropic and inflammation factors (Li et al., 2023). One study reported that a 24 − week Tai Chi training program improved global cognitive function, cognitive processing speed, executive function, attention, and memory function in older adults with cognitive decline (Shurui, 2022).

Previous meta-analyses have suggested that Tai Chi can have beneficial effects for patients with MCI, but that some meta-analyses have involved limitations (Jianguo et al., 2017; Zheng et al., 2017). For example, in a meta-analysis by Zhang, the samples of previous studies were reported to be mixed, including healthy individuals, patients with Alzheimer’s disease, and patients with MCI (Jianguo et al., 2017). In a meta-analysis by Zheng, the designs of previous studies were found to be inconsistent, including descriptive analyses and randomized controlled trials (RCTs) (Zheng et al., 2017). Significantly, until the present study, there had been no systematic review and meta−analysis of RCTs to allow the efficacy of Tai Chi treating MCI to be verified. So, in the current study, we performed a systematic review and meta−analysis of secondary data from RCTs and focused on patients with MCI to provide the efficacy of Tai Chi more reliable evidence.

## 2 Methods

### 2.1 Study design and protocol registration

The review’s protocol followed the Preferred Reporting Items for Systematic Review and Meta−Analysis (PRISMA). The meta−analysis was performed following the statement of the PRISMA guidelines (Liberati et al., 2009) and Cochrane Collaboration handbook (Cumpston et al., 2019) in order to provide comprehensive and transparent reporting of methods and results. This study has been duly registered at the International Prospective Register of Systematic Reviews under registration number CRD42024558313.

### 2.2 Search strategy

We conducted an extensive search across various databases, including CNKI, CBM, Wanfang Data, VIP, PubMed, Embase, Duxiu Database, Web of Science and Cochrane Library until April 2024. Meanwhile, we also searched other databases such as Open Grey, clinicaltrials.gov and WHO Clinical Trial Registration Center. We further searched for other identified potential studies from the previous meta−analysis. Randomized controlled trials meeting the inclusion criteria were then manually screened. The search strategy for PubMed is presented in Table 1.

**Table 1:** Search strategy used in PubMed.

Number	Search terms
#1	“tai ji”[Title/Abstract] OR “Tai Chi”[Title/Abstract] OR “Tai Ji Quan”[Title/Abstract] OR “Taiji”[Title/Abstract] OR “Taijiquan”[Title/Abstract] OR “Taichi”[Title/Abstract] OR “Tai Chi Chuan”[Title/Abstract] OR “TCC”[Title/Abstract]
#2	mild cognitive impairment[MeSH Terms]
#3	“mild cognitive impairments”[Title/Abstract] OR “cognitive impairments mild”[Title/Abstract] OR “cognitive declines”[Title/Abstract] OR “neurocognitive disorders”[Title/Abstract] OR “neurocognitive disorders”[Title/Abstract] OR “mild neurocognitive disorder”[Title/Abstract] OR “MCI”[Title/Abstract] OR “Mild Cognitive Impairments”[Title/Abstract] OR “Cognitive Impairment, Mild”[Title/Abstract] OR “Cognitive Impairments, Mild[Title/Abstract] OR “Impairment, Mild Cognitive[Title/Abstract]OR “Impairments, Mild Cognitive[Title/Abstract]
#4	#2 AND #3
#5	Randomized controlled trial[MeSH Terms]
#6	(((((randomly) OR (randomized)) OR (RCT)) OR (Randomizedclinical trials)) OR (trials)) OR (Random allocation)
#7	#5 AND #6
#8	#1 AND #4 AND #7

### 2.3 Study eligibility criteria

#### 2.3.1 Inclusion criteria

1. Study type: Randomized controlled trial.
2. Inclusion population: Patients with cognitive impairment meeting the internationally recognized diagnostic criteria for cognitive dysfunction (Anand and Schoo, 2024) and no comorbidities. And the included studies used MoCA, DRT, VFT, and TMT as outcome indicators.
3. Intervention methods: the intervention group is Tai Chi practice, and there are no specific restrictions on the practice plan of Tai Chi.
4. The published languages of the literature are Chinese and English, and the full text is available.

#### 2.3.2 Exclusion criteria

1. Repeated publications, poor quality assessment, and unavailability of full−text literature.
2. Literature with failure of randomization and significant differences in baseline data between groups.

### 2.4 Data extraction

Two independent investigators (XXS and LJP) read the title, abstract and full text. According to inclusion and exclusion criteria, they screened the literature and cross−checked the results. Results Data were extracted separately by a third investigator (XRL). If there was a disagreement, a fourth investigator (XYZ) will be consulted. Data extracted from the included literature included the following: first author, time of publication, mean age or age range, study sample size, intervention and control groups taken, duration, and outcomes.

### 2.5 Outcomes

The primary outcome was MoCA. Secondary outcomes were DRT, VFT and TMT.

### 2.6 Quality assessment

The quality of the included studies was assessed by the revised Cochrane risk of bias tool (ROB 2.0) in the Cochrane Handbook for Systematic Reviews of experiments. The following parameters were evaluated: randomization process, deviations from intended experiments, missing outcome data, measurement of the outcome, and selection of the reported result. Each item was determined to be at high risk of bias, some concerns (unclear risk of bias), or low risk of bias. The combined evaluation of the above items resulted in overall bias.

### 2.7 Quantitative synthesis

Stata 15.0 software was used to conduct the meta-analysis of the selected studies. The mean difference (MD) was taken as continuous effect size indicators for variables, using 95% confidence intervals (95%CI) in forest maps. When the same intervention effect is measured by different methods or units, the standardized mean difference (SMD) is the appropriate choice as the combined statistic. Data were pooled using fixed-effects model or random-effects model to determine SMD and 95% CI. If the heterogeneity test is *I*^2^ > 50%, the random-effects model was adopted. If the heterogeneity test is *I*^2^ < 50% and *p* > 0.10, fixed-effects model was adopted. In case of high heterogeneity (*I*^2^ > 50%), sensitivity analysis and subgroup analysis were used to analyze sources of heterogeneity. For sensitivity analysis, it could help find the source of heterogeneity by re−estimating the combined effect using the one−by−one elimination method. For subgroup analysis, studies could be divided into different subgroups based on Tai Chi types, and whether subgroup factors could be proved to be the source of heterogeneity. Statistically significant was considered for outcomes with a *p* < 0.05.

## 3 Result

### 3.1 Literature screening process

**Figure 1:**
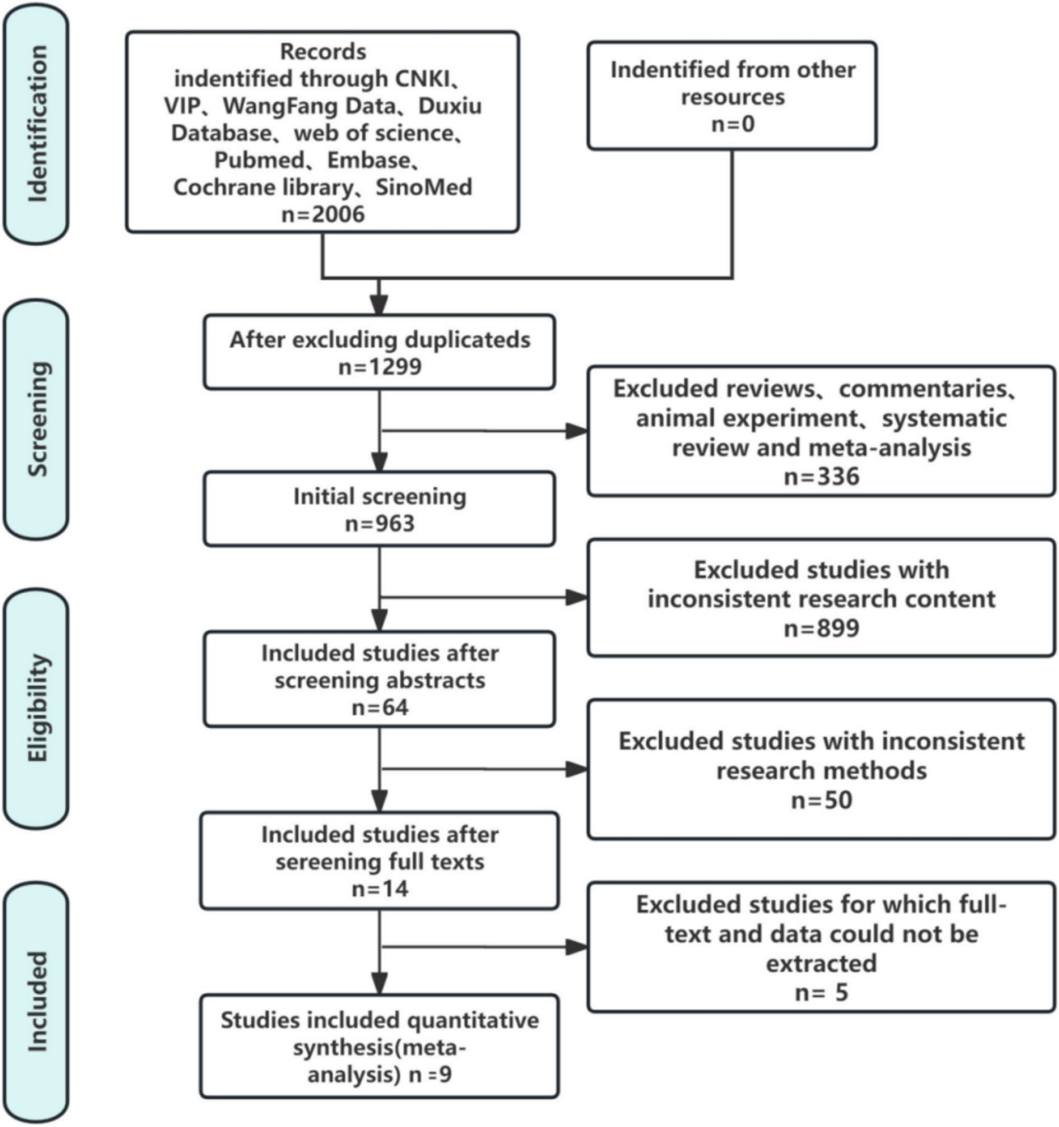
Flowchart with the search results and selection details.

The search process yielded 2006 articles. There were 324 articles from Pubmed, 270 articles from Embase, 189 articles from Cochrane Library, 306 articles from Web of Science, 331 articles from CNKI, 250 articles from Wangfang Data, 220 articles from VIP, 44 articles from CBM, 72 articles from Duxiu Database. Two researchers independently screened these articles, obtained 1,299 articles after Noteexpress review, 963 articles after excluding reviews, commentaries, animal experiments, systematic reviews and meta−analysis. Then there were 64 articles after excluding inconsistent research content, 14 articles after excluding inconsistent research methods, 5 research articles that cannot extract full text and data. Finally, 9 studies (Bao and Liu, 2019; Lam et al., 2012; Lam et al., 2012; Li F. Z. et al., 2022; Liu et al., 2022; Sungkarat et al., 2017; Sungkarat et al., 2018; Tian, 2019; Yu et al., 2022) met the inclusion criteria. Figure 1 illustrated the specific retrieval process.

### 3.2 Basic characteristics of included studies

There were 9 studies with a total of 1,046 participants. Table 2 presented the main characteristics of interventions and participants. The included studies comprised 461 participants in 9 intervention groups and 585 participants in 9 control groups. All intervention groups in the study involved one of the following Tai Chi types: 24-forms Tai Chi, 8-forms Tai Chi and 10-forms Tai Chi. The intervention duration ranged from 8 to 48 weeks. Five studies used the MoCA (Bao and Liu, 2019; Li F. Z. et al., 2022; Liu et al., 2022; Tian, 2019; Yu et al., 2022) as the primary outcome measure. Five studies provided data for the DRT (Lam et al., 2012; Lam et al., 2012; Liu et al., 2022; Sungkarat et al., 2018; Yu et al., 2022), 4 studies provided data for the VFT (Lam et al., 2012; Li F. Z. et al., 2022; Yu et al., 2022) and 6 studies provided data for the TMT (Li F. Z. et al., 2022; Liu et al., 2022; Sungkarat et al., 2017; Sungkarat et al., 2018; Tian, 2019; Yu et al., 2022) as the secondary outcome measures. Out of the studies analyzed, all 9 studies was designed for men and women. The participants’ mean age ranged from 64.90 to 85.00 years. Among the 9 intervention groups, 5 involved 24-forms Tai Chi, 2 involved 8-forms Tai Chi and 2 involved 10-forms Tai Chi. The frequency of weekly interventions ranged from 2 to 4 times per week. The session duration ranged from 30 to 60 min. Finally, the weekly time spent on interventions ranged from 90 to 240 min.

**Table 2:** Summary of the main characteristics.

Study	Population (T/C)	Country	Age	Intervention	Control	Minutes per session (min)	frequency (times/ week)	Duration	Randomization sequence generation	Blinding	Outcome	Completeness
Bao NN 2019 (Bao and Liu, 2019)	31/31	China	T:68.22 ± 9.8 C:65.62 ± 9.34	24 − forms Tai Chi	Health education	30	3	24 weeks	Random number table	No blind	(1)	Yes
Lam LC 2012 (Lam et al., 2012)	171/218	China	T:77.2 ± 6.3C:78.3 ± 6.6	24 − forms Tai Chi	Stretching and conditioning training	≥30	≥3	48 weeks	Randomized control (but does not describe random allocation method)	single blind	(2)(3)	Yes
Lam LC 2014 (Lam et al., 2014)	96/169	China	T:77.2 ± 6.3C:78.3 ± 6.6	24 − forms Tai Chi	Stretching and relaxation exercises	≥30	≥3	12 weeks	Randomized control (but does not describe random allocation method)	single blind	(2)(3)	Yes
Li FZ 2022 (Li et al., 2022)	24/22	America	T:74.5 ± 5.6C:74.9 ± 6.3	8-forms Tai Chi	Stretching	60	2	16 weeks	Random number table	single blind	(1)(3)(4)	Yes
Liu CL 2022 (Liu et al., 2022)	17/17	China	T:73.2 ± 6.3C:73.4 ± 6.5	24-forms Tai Chi	No intervention	50	3	12 weeks	Randomized control (but does not describe random allocation method)	single blind	(1)(2)(4)	Yes
Somporn S 2017 (Sungkarat et al., 2017)	33/33	Thailand	T:68.3 ± 6.7C:67.5 ± 7.3	10-forms Tai Chi	Health education	50	3	12 weeks	Random number table	Single blind	(4)	Yes
Somporn S 2018 (Sungkarat et al., 2018)	33/33	Thailand	NA	10-forms Tai Chi	Health education	50	3	24 weeks	Random number table	single blind	2()(4)	Yes
Tian L 2020 (Tian, 2019)	46/50	China	65 to 85	8-forms Tai Chi	No intervention	60	4	16 weeks	Randomized control (but does not describe random allocation method)	No blind	(1)(4)	Yes
Yu AP 2022 (Yu et al., 2022)	10/12	China	T:64.90 ± 3.37C:66.86 ± 5.74	24-forms Tai Chi	Brisk walking	60	3	8 weeks	Random number table	single blind	(1)(2)(3)(4)	Yes

T, treatment group; C, control group; (1) MoCA, Montreal cognitive assessment; (2) DRT, delayed recall test; (3) VFT, verbal fluency test; (4) TMT, Trail Making Test.

### 3.3 Quality assessment

**Figure 2:**
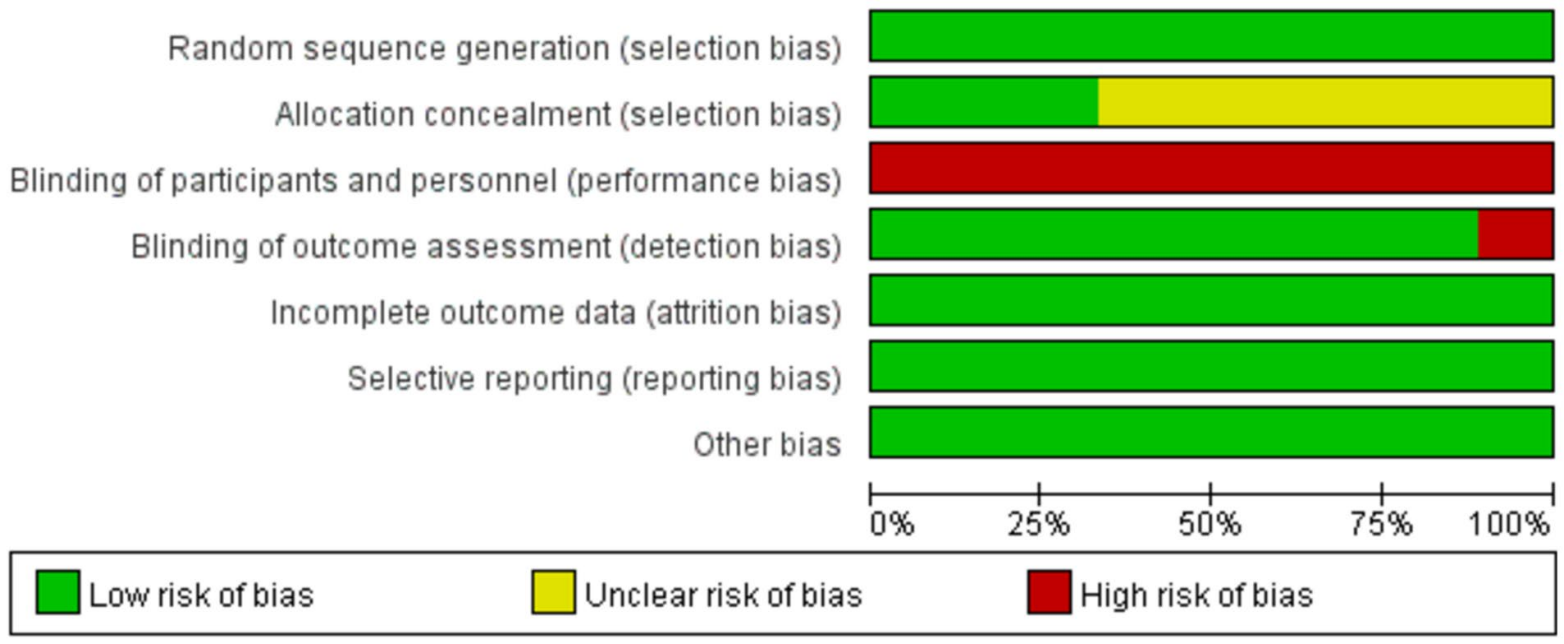
Risk of bias graph.

The methodological evaluation of the quality of the included literature was as shown in Figure 2. All the 9 studies reached a low risk of bias and had a high quality. For the random allocation method, all studies mentioned random allocation, of which 5 studies mentioned the use of random number table allocation. For allocation concealment, 6 studies were considered to be risk fuzzy, and 3 studies were considered to be low risk. For outcome data integrity bias, all studies were considered to be low−risk. All 9 studies were considered to be low−risk for bias in selective reporting results. For detection bias, 8 studies were considered to be low risk, and 1 study was considered to be high risk. For the blinding of participants, all studies were considered to be high−risk. Due to the particularity of Tai Chi, the subjects must participate in the exercise, so the blind method of the subjects was difficult to achieve. Therefore, the lack of blinding was not considered to be the cause of bias.

Biased items include:

1. Randomization was reported, but some did not specifically describe the randomization process;
2. Whether allocation concealment was implemented in the randomization scheme, some projects were not reported or unclear;
3. Some studies did not explicitly report whether blinding was implemented;
4. Whether the registration of some research programs had not been reported or the discussion of the reported items was not clear.

### 3.4 Meta analysis results

#### 3.4.1 Effects of Tai Chi on MoCA in MCI patients

**Figure 3:**
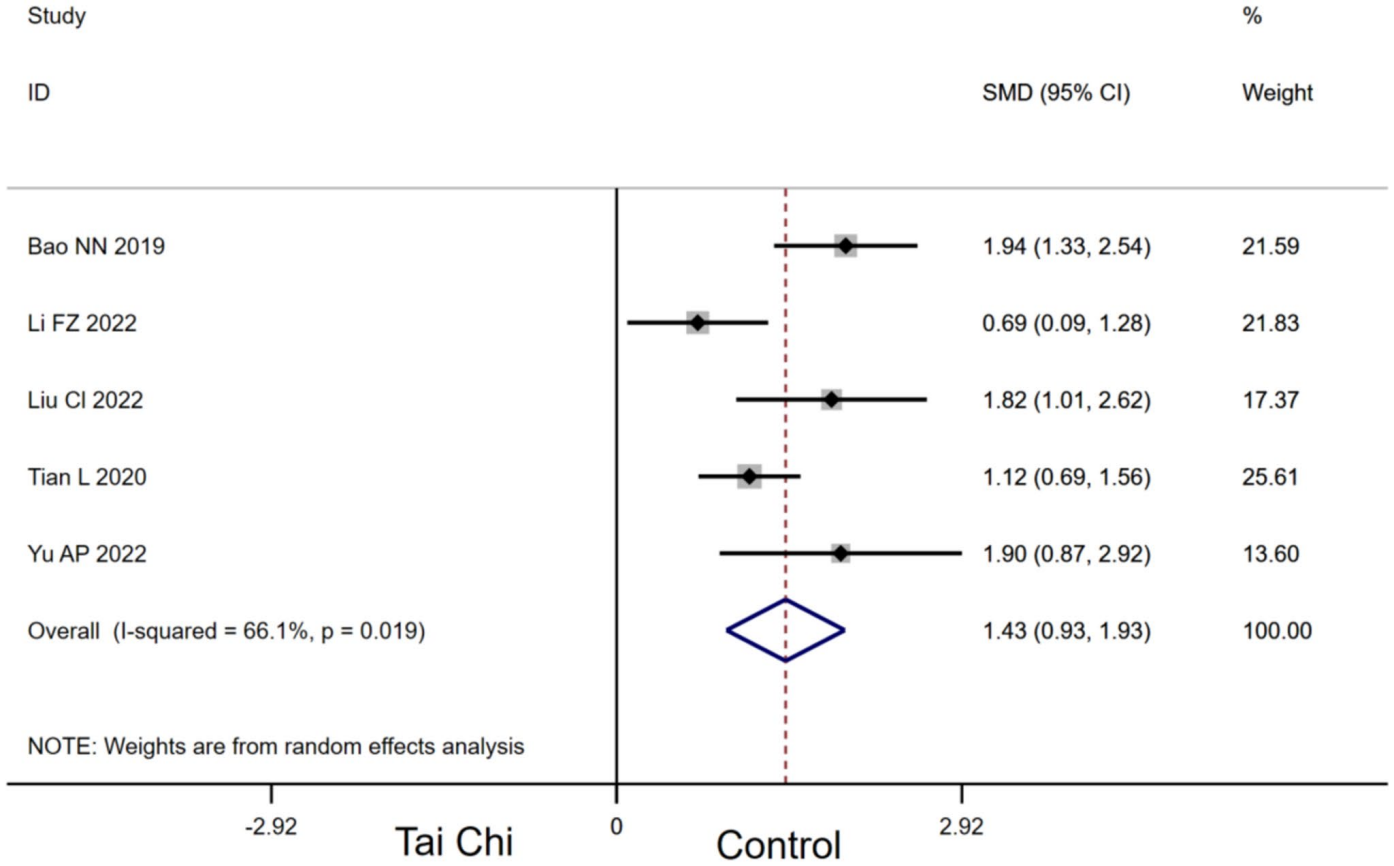
Forest plots of effects of Tai Chi on MoCA in MCI patients.

Five studies provided data for MoCA. Compared with the control group, Tai Chi had a significant effect on improving MoCA in MCI patients [SMD, 1.43 (95% CI, 0.93 to 1.93), *p* < 0.00001, *I*^2^ = 66.1%, Figure 3].

#### 3.4.2 Effects of Tai Chi on DRT in MCI patients

**Figure 4:**
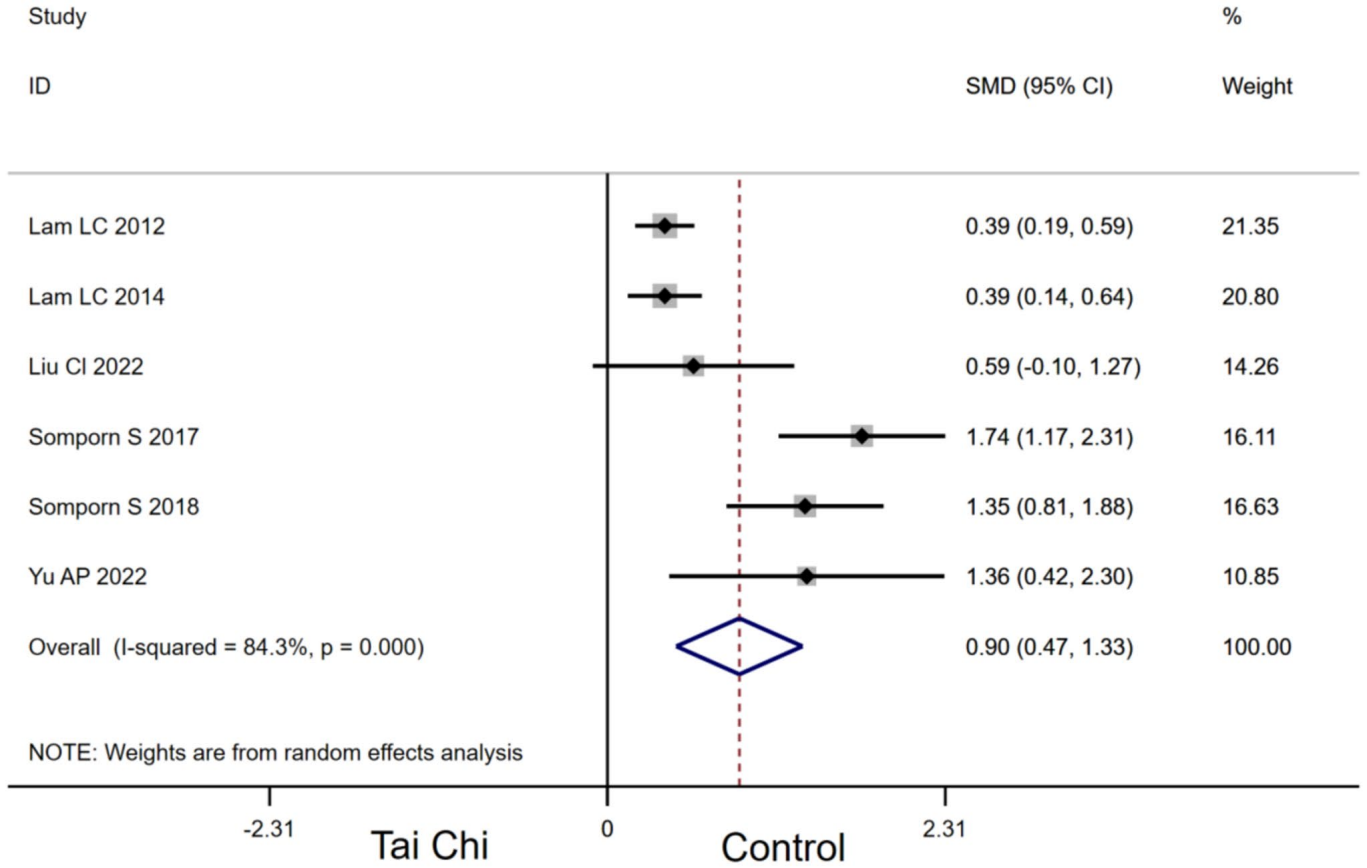
Forest plots of effects of Tai Chi on DRT in MCI patients.

Six studies provided data for DRT. Compared with the control group, Tai Chi had a significant effect on improving DRT in MCI patients [SMD, 0.90 (95% CI, 0.47 to 1.33), *p* < 0.00001, *I*^2^ = 84.3%, Figure 4].

#### 3.4.3 Effects of Tai Chi on VFT in MCI patients

**Figure 5:**
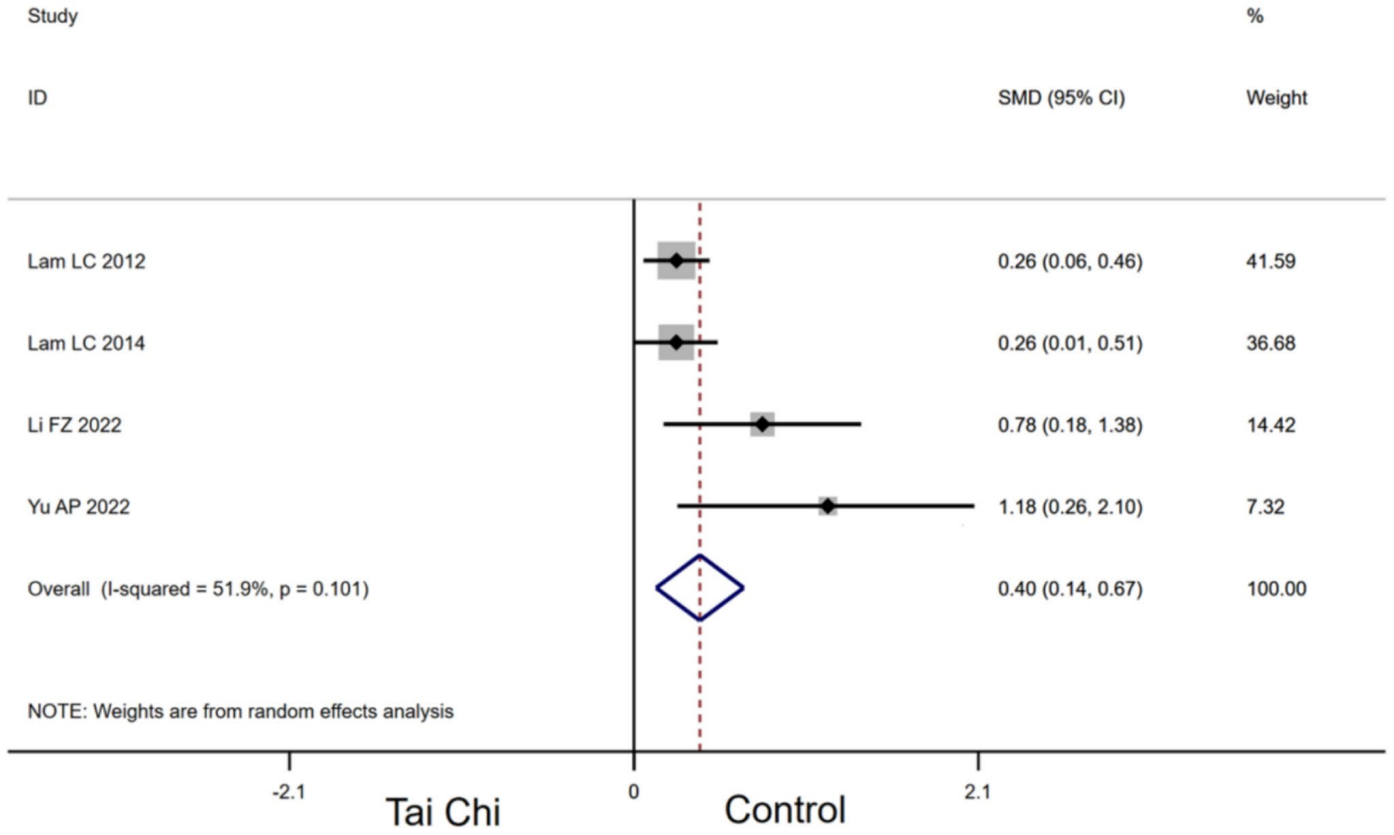
Forest plots of effects of Tai Chi on VFT in MCI patients.

Four studies provided data for VFT. Compared with the control group, Tai Chi had a significant effect on improving VFT in MCI patients [SMD, 0.40 (95% CI, 0.14 to 0.67), *p* < 0.00001, *I*^2^ = 51.9%, Figure 5].

#### 3.4.4 Effects of Tai Chi on TMT in MCI patients

**Figure 6:**
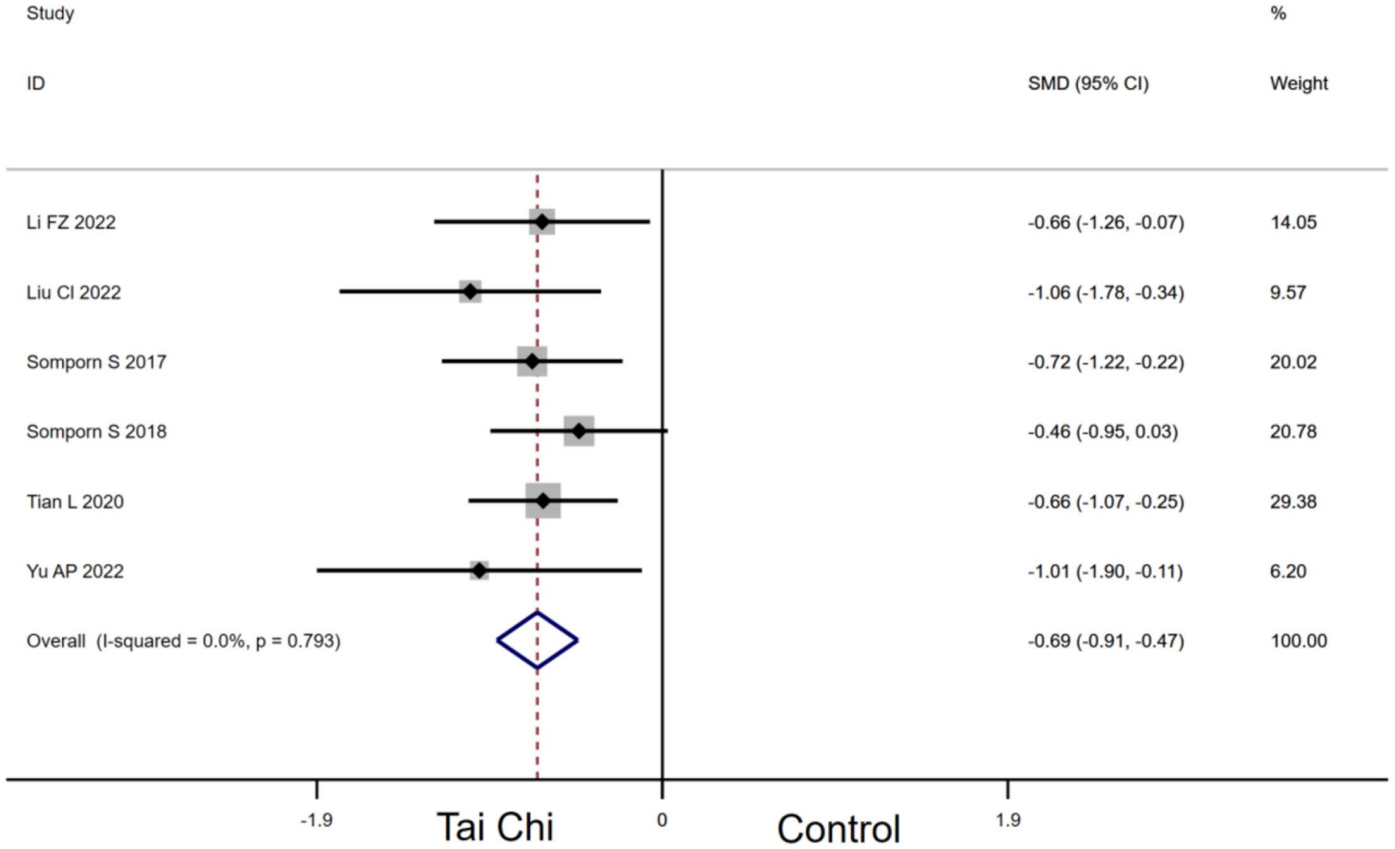
Forest plots of effects of Tai Chi on TMT in MCI patients.

Six studies provided data for TMT. Compared with the control group, Tai Chi had a significant effect on improving TMT in MCI patients [SMD, −0.69 (95% CI, −0.91 to −0.47), *p* < 0.00001, *I*^2^ = 0.0%, Figure 6].

#### 3.4.5 Subgroup analysis

**Figure 7:**
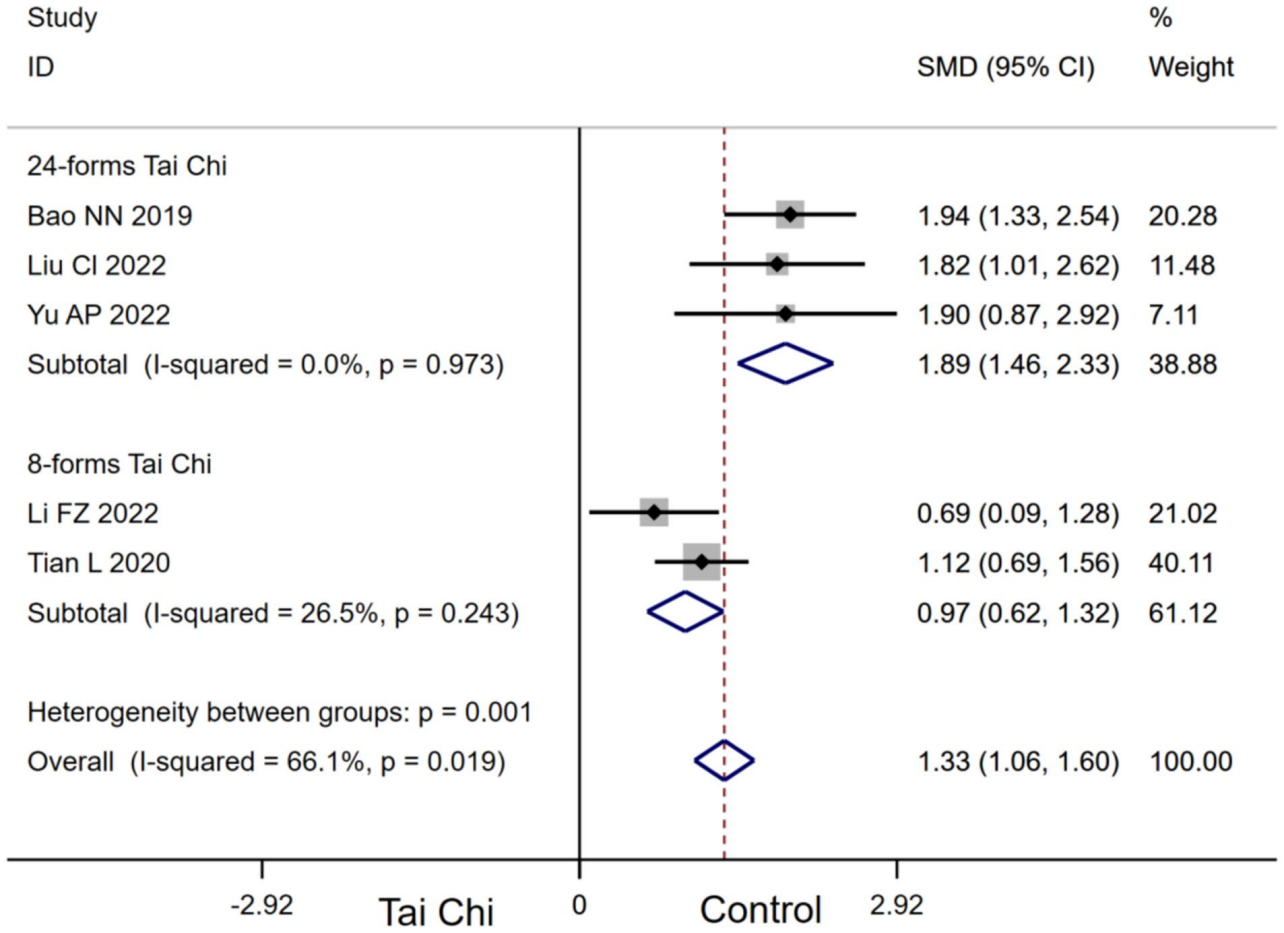
Subgroup analysis of the effect of Tai Chi types on MoCA in MCI patients.

Stratifying the analysis by Tai Chi types, 24-forms Tai Chi [SMD, 1.89 (95%CI, 1.46 to 2.33), *I*^2^ = 0.0%, *p* < 0.00001] and 8-forms Tai Chi [SMD, 0.97 (95%CI, 0.62 to 1.32), *I*^2^ = 26.5%, *p* < 0.00001, Figure 7] significantly improved MoCA in MCI patients. Subgroup analysis indicated that 24-forms Tai Chi had a greater effect on improving MoCA in MCI patients.

**Figure 8:**
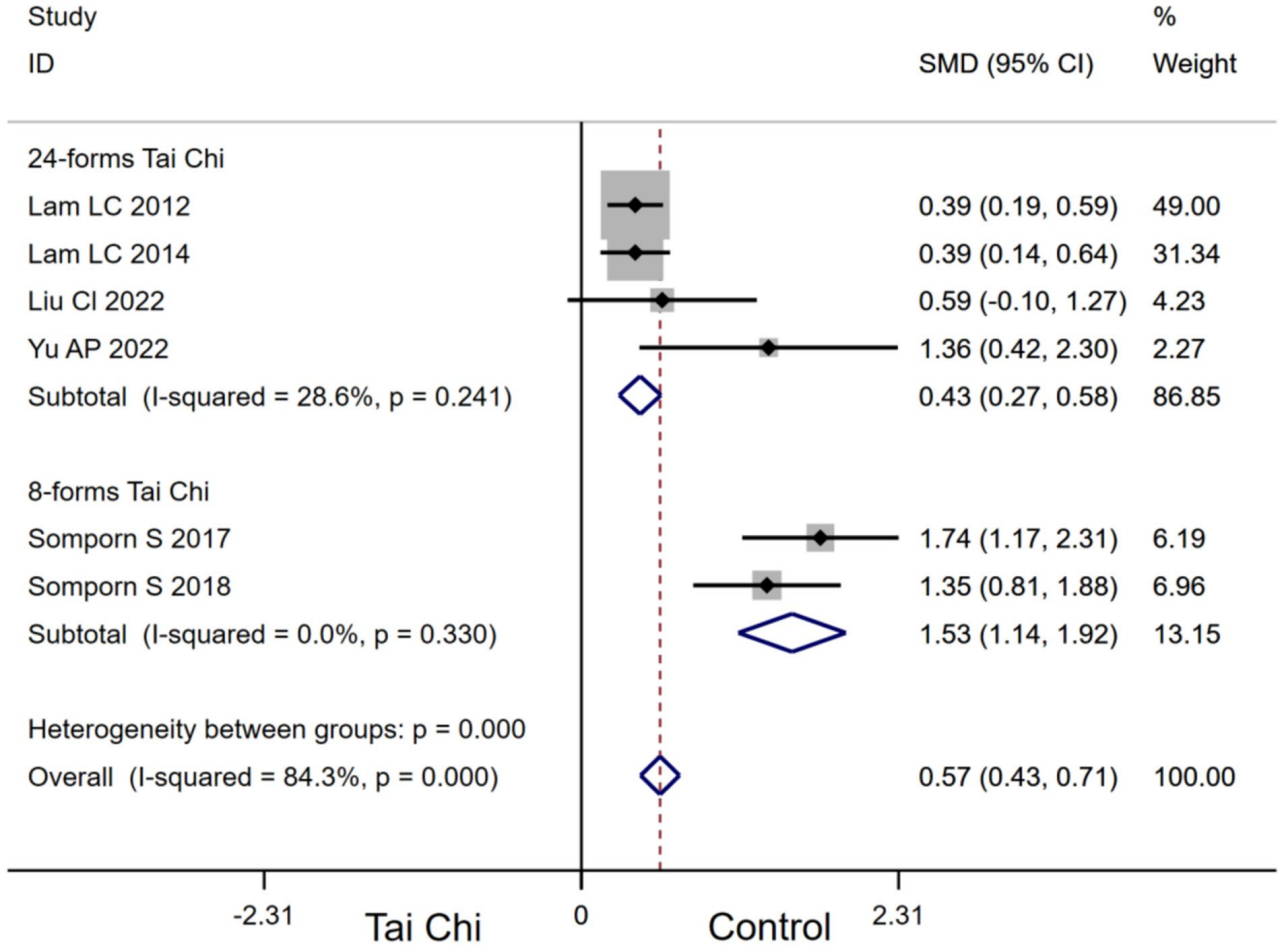
Subgroup analysis of the effect of Tai Chi types on DRT in MCI patients.

In addition, stratifying the analysis by Tai Chi types, 24-forms Tai Chi [SMD, 0.43 (95%CI, 0.27 to 0.58), *I*^2^ = 28.6%, *p* < 0.00001] and 10-forms Tai Chi [SMD, 1.53 (95%CI, 1.14 to 1.92), *I*^2^ = 0.0%, *p* < 0.00001, Figure 8] significantly improved DRT in MCI patients. Subgroup analysis indicated that 10-forms Tai Chi had a greater effect on improving DRT in MCI patients.

#### 3.4.6 Sensitivity analysis

**Figure 9:**
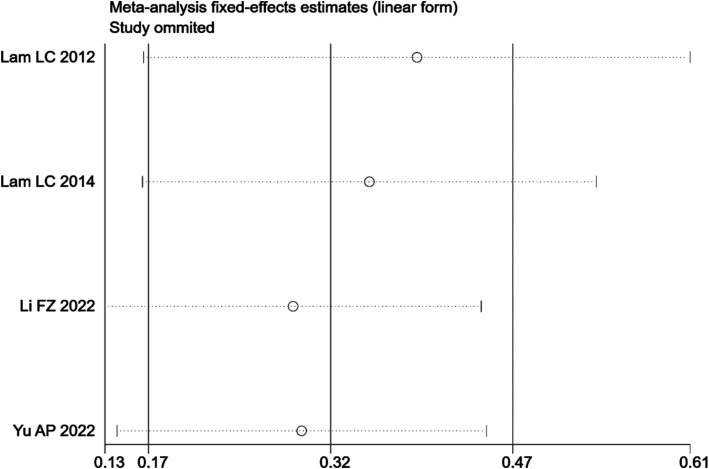
Sensitivity analysis results of VFT.

Sensitivity analyses revealed that the overall effect of Tai Chi on VFT (Figure 9) in MCI patients remained consistent in terms of direction and compatibility levels when any of the included studies were omitted.

#### 3.4.7 Publication bias

**Figure 10:**
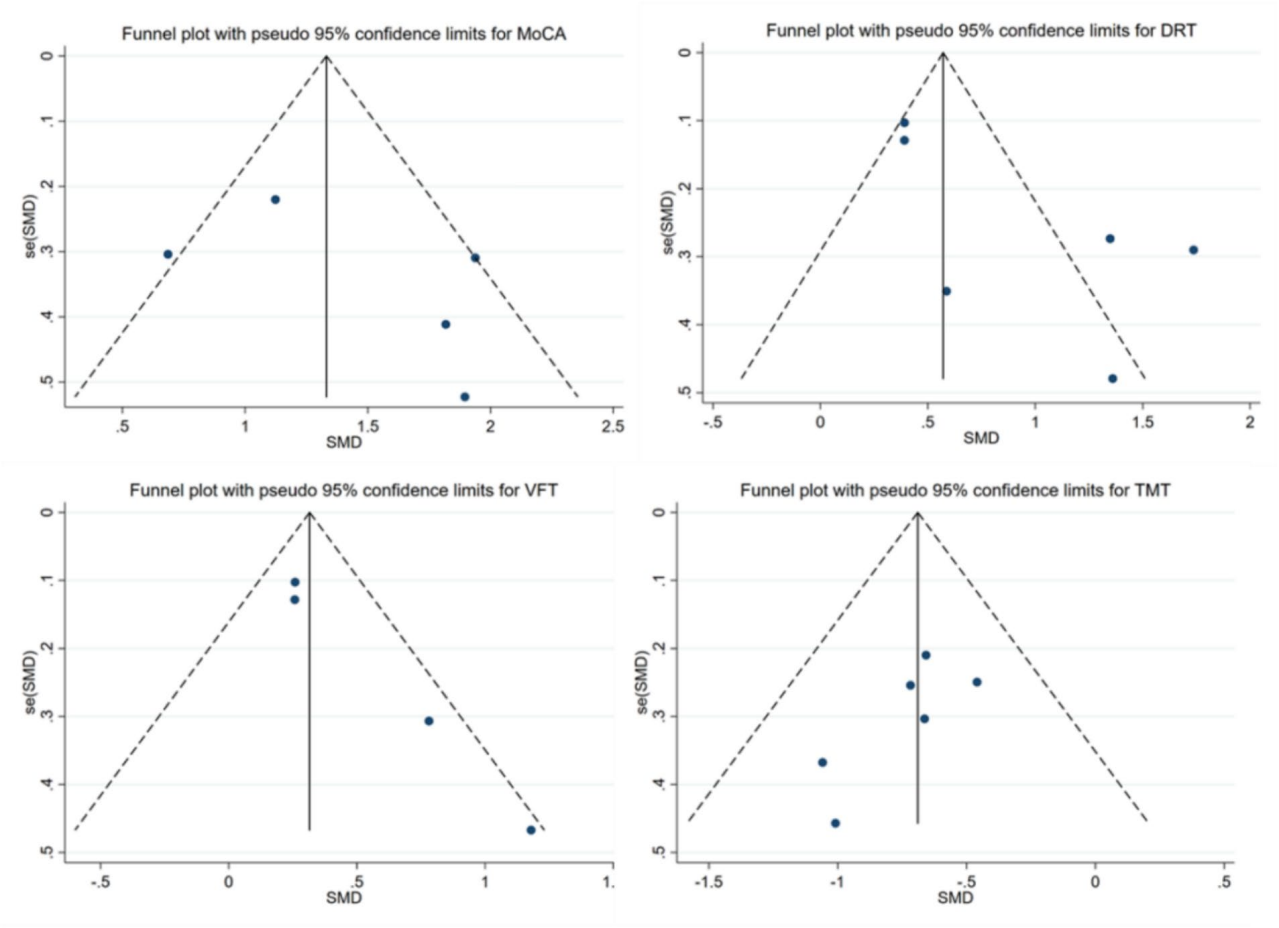
Funnel plot.

To determine if there is any bias in the publications, we conducted the funnel plot (Figure 10). Visual inspection of the funnel plot suggested the absence of funnel plot asymmetry. Based on the results of Begg’s test, small sample size studies did not significantly influence the results of MoCA (*p* = 0.806), DRT (*p* = 0.260), VFT (*p* = 0.089) and TMT (*p* = 0.133).

#### 3.4.8 Grading of the quality of evidence

The quality of evidence was assessed using the GRADE (Grades of Recommendation, Assessment, Development, and Evaluation) method and was categorized into high, moderate, low, or very low grade. The assessment criteria employed in the GRADE method encompassed risk of bias, inconsistency, indirectness, imprecision, and publication bias. A specific grade was assigned to each outcome. The results for GRADE in this study are shown in Table 3.

**Table 3:** Grading of the quality of evidence.

Certainty assessment							Number of patients		Effect	Certainty	Importance
Outcome of studies	Study design	Risk of bias	Inconsistency	Indirectness	Imprecision	Other considerations	Intervention group	Control group	Absolute (95% CI)		
MoCA	Randomised not seriousnonetrials	Serious^a^	Serious^b^	Not serious	Not serious	None	128	132	SMD 1.43(0.93 to 1.93)	“ФФOO low”	Important
DRT	Randomised not seriousnonetrials	Serious^a^	Serious^b^	Not serious	Not serious	None	327	449	SMD 0.90(0.47 to 1.33)	“ФФOO low”	Important
VFT	Randomised not seriousnonetrials	Serious^a^	Serious^b^	Not serious	Not serious	None	301	399	SMD 0.40(0.14 to 0.67)	“ФФOO low”	Important
TMT	Randomised not seriousnonetrials	Serious^a^	Serious^b^	Not serious	Not serious	None	163	167	SMD −0.69(−0.91 to −0.47)	“ФФOO low”	Important

Montreal Cognitive Assessment (MoCA); Secondary outcomes were Delayed Recall Test (DRT); verbal fluency test (VFT); Trail Making Test (TMT). ^a^Lacking blinding or unclear allocation. ^b^Substantial heterogeneity. ^c^Small sample size or only a few included articles. Ф Represents the evidence quality rating; ФФ is low; ФФOO means “low” certainty.

## 4 Discussion

### 4.1 Main findings

The purpose of this study was to examine the effectiveness of Tai Chi on cognitive function in MCI patients. Nine studies were included and the findings indicated that Tai Chi significantly improved cognitive function in MCI patients. Subgroup analyses showed that 24-forms Tai Chi was more effective than 8-forms Tai Chi for improving MoCA in MCI patients, whereas 10-forms Tai Chi was more effective than 24-forms Tai Chi for improving DRT in MCI patients.

### 4.2 Effects of Tai Chi on cognitive function in MCI patients

This systematic review and meta-analysis suggested that Tai Chi holds the potential to improve cognitive function in MCI patients, which could be evidenced by improvements in MoCA, DRT, VFT and TMT. Commonly, the MoCA is used to assess the global cognitive function, the DRT is used to assess memory function, the VFT is used to assess language fluency and the TMT is used to assess executive function.

Our study proved that Tai Chi was suggested to improve MoCA in MCI patients, enhancing the global cognitive function (Jasim et al., 2023). The general mechanism can be explained as follows. Firstly, accumulating neuroimaging studies have demonstrated that MCI is a disorder characterized by brain dysconnectivity. Declines in the hippocampus and the medial prefrontal cortex manifest as disrupted connectivity among brain regions (Marshall et al., 2011). Previous studies reported significant increases in resting-state functional connectivity (rs-FC) (Jasim et al., 2023) between the medial prefrontal cortex and bilateral hippocampus (Li et al., 2014), and between the medial prefrontal cortex and medial temporal lobe (Tao et al., 2017b) after Tai Chi training. Tai Chi increases rs-FC between left middle frontal gyrus and left superior parietal lobule (Cui et al., 2019), alters resting−state synchrony, and causes more efficient patterns of brain activation (Chaddock-Heyman et al., 2013), so neural activity associated with cognitive control is changed to improve global cognitive function. In addition, studies reported that 12 weeks of Tai Chi training improved cognitive function in patients with MCI and increased the rs-FC between the hippocampus and medial prefrontal cortex, while decreasing the functional connectivity among the dorsolateral prefrontal cortex, left superior frontal gyrus, and anterior cingulate gyrus (Tao et al., 2017a; Meng et al., 2023). Therefore, the beneficial effects of Tai Chi on cognitive functions in older patients with MCI might be mediated by altering the connectivity of the brain network. Secondly, Brain−derived neurotrophic factor (BDNF) is a protein that participates in the differentiation, growth, damage repair, the occurrence of new synapses (Colucci D’Amato et al., 2020), and crucial for the regulation of brain plasticity and memory function (Liqin et al., 2023). Tai Chi improves memory functions by upregulating plasma BDNF (Li et al., 2024), altering the hippocampus at structural and functional levels (Yue et al., 2020) and activating the prefrontal cortex and improving the synaptic plasticity (Wu et al., 2018). Thirdly, MCI is also caused by abnormal metabolism of neurochemicals in the brain. The hallmark neuropathological substrates for MCI are *β*−amyloid (Aβ) plaques and intracellular Tau neurofibrillary tangles (Chandra et al., 2019). The reduction in Aβ1–42 is considered as evidence of amyloid deposition in the brain. An RCT conducted in China reported that multimodal exercises increased Aβ1-42 in peripheral serum, and decreased Tau protein. Additionally, changes in cognitive function were negatively associated with changes in plasma Aβ1-42 and Tau protein content (Liqin et al., 2023). Tai Chi is a type of multimodal exercises and may have a similar effect.

Tai Chi has been suggested to significantly improve DRT in MCI patients to strengthen memory. Memory decline is the most dominant and common clinical presentation in patients with MCI. Tai Chi enhances the hippocampal plasticity to improve delay recall (Lin M. et al., 2024). Additionally, studies have found that neurochemicals, including *N*−acetyl aspartate (NAA), choline, creatine (Cr), and glutamate and glutamine, have abnormal metabolic changes in the pathological process of MCI. Of which, the level of NAA is closely associated with cognitive dysfunction, especially memory impairment (Valatkevičienė et al., 2023). A previous study indicated that 12 weeks of Tai Chi training significantly increases NAA/Cr ratios in posterior cingulate gyrus and promotes neuronal integrity and viability (Zhou et al., 2018). An increased NAA/Cr ratio may suggest an increase in neuroplasticity.

In our study, Tai Chi has been suggested to significantly improve VFT in MCI patients to strengthen language fluency. Consensus has been reached that both semantic and phonemic fluency tasks are impaired in neurodegenerative disorders (Fama et al., 2000)^.^ Compared with the MoCA, verbal fluency measures may be sensitive to more subtle declines found in a−MCI (Rinehardt et al., 2014; Price et al., 2012). Mind–body exercises were beneficial for increasing the fluency of verbal language and promoting learning ability, especially Tai Chi (Wu et al., 2019). Eight weeks of Tai Chi increases the gray matter volume in the left middle occipital gyrus, left superior temporal gyrus, and right middle temporal gyrus (Cui et al., 2019). These regions are highly correlated with visual information processing, emotion regulation, language/semantic processing, and memory encoding and retrieval (Liu et al., 2021). Additionally, research suggests that the long−term practice of Tai Chi improves the regulatory function of the autonomic nervous system in MCI patients, promoted nerve connection, and increases the brain’s information processing capability (Solianik et al., 2021), and thus improved MCI patients’ language. Similar to our findings (in Figure 5), a previous study reported that the Tai Chi group’s evaluation result of language fluency was obviously better than the control group (Hawkes et al., 2014b).

Our findings showed that Tai Chi decreases TMT in MCI patients (in Figure 6). The results suggested that Tai Chi may improve MCI patients’ executive function, which is consistent with other meta−analyses (Wayne et al., 2014; Chen et al., 2020). Tai Chi demonstrated larger P300 event-related potential in switch trial amplitudes compared to sedentary controls, suggesting beneficial effects on the neural substrates of executive function (Hawkes et al., 2014a). The P300 event-related potentia is a neuroelectric index of human executive function. Moreover, study results suggest that Tai Chi practice may activate the prefrontal cortex and improve working memory, attentional focus, and processing speed through concurrent physical and mental activity (Qi et al., 2023). This may be one mechanism for the improvement in executive function tests found after Tai Chi training.

### 4.3 Subgroup analysis

Tai Chi significantly improved MoCA (*I*^2^ = 66.1%) and DRT (*I*^2^ = 84.3%) in MCI patients with high heterogeneity between groups, indicating disparities in the outcomes of the included studies. Consequently, we conducted subgroup analyses of MoCA and DRT metrics, indicating that Tai Chi type may be an influencing factor for the effect of Tai Chi on improving MoCA and DRT in MCI patients. The results of our meta-analysis indicated that 24-forms Tai Chi was the most effective Tai Chi type for improving MoCA in MCI patients. 24-forms Tai Chi was developed by the China National Sports Commission on the basis of Yang style Tai Chi 1956 (Lin J. et al., 2024). 24-forms Tai Chi is one of the more systematic Tai Chi types, reflecting the full movement characteristics of Tai Chi. Wei found that 24-forms Tai Chi directly optimized the neurocognitive mechanism of MCI patients by activating related brain areas and strengthening brain area connectivity to comprehensively promote the global cognitive function plasticity of MCI patients (Wei, 2024). Regarding DRT metrics, the results indicated that 10-forms Tai Chi may be the most effective Tai Chi type for improving DRT in MCI patients. It was found that the effects of 10-forms Tai Chi specifically improved memory function in the cognitive domain (Sungkarat et al., 2017). This effect may be related to the task-specific requirements of 10-forms Tai Chi, such as movement recall, movement switching, and movement planning processing (Song et al., 2021).

## 5 Limitations and perspectives

There are some limitations in the published meta−analysis articles about the effectiveness of Tai Chi in cognitive impairment, as follows: All the articles included in our research are RCTs and their subjects are patients with cognitive impairment. These high−quality research data can improve the level of evidence. Thus, our research provided more reliable evidence−based medical evidence for MCI.

Meanwhile, it is suggested to make a network meta−analysis about the effectiveness of different types of Tai Chi for MCI patients in the future to provide a reference for selecting appropriate Tai Chi type. It could help enhance the patients’ quality of life.

## 6 Conclusion

In conclusion, Tai Chi improved the MCI patients’ cognitive function including the global cognitive function, memory function, language fluency, executive function. Tai Chi types might be the influence factor on Tai Chi improving the global cognitive function and memory function in MCI patients.

## Data Availability

The original contributions presented in the study are included in the article/supplementary material, further inquiries can be directed to the corresponding author.
